# Texting for life: a mobile phone application to connect pregnant women with emergency transport and obstetric care in rural Nigeria

**DOI:** 10.1186/s12884-023-05424-9

**Published:** 2023-03-08

**Authors:** Friday Okonofua, Lorretta Ntoimo, Ermel Johnson, Issiaka Sombie, Solanke Ojuolape, Brian Igboin, Wilson Imongan, Chioma Ekwo, Ogochukwu Udenigwe, Sanni Yaya, Anne B. Wallis, Joy Adeniran

**Affiliations:** 1 Women’s Health and Action Research Centre (WHARC), Km 11 Benin-Lagos Expressway, Igue-Iheya, Benin City, Edo State Nigeria; 2 Centre Leader, Centre of Excellence in Reproductive Health Innovation, University of Benin, Benin City, Nigeria; 3 Department of Obstetrics and Gynaecology, University of Benin and University of Benin Teaching Hospital, Benin City, Nigeria; 4 Department of Demography and Social Statistics, Federal University Oye-Ekiti, Oye-Ekiti, Nigeria; 5 West African Health Organization, Bobo-Dioulasso, Burkina Faso; 6 Federal Ministry of Health, Abuja, Nigeria; 7 School of International Development and Global Studies, University of Ottawa, Ottawa, Canada; 8 The George Institute for Global Health, University of Oxford, Oxford, UK; 9 Department of Epidemiology, School of Public Health & Information Sciences, University of Louisville, Louisville, USA; 10 Berth Technologies, Lagos, Nigeria

**Keywords:** Maternal mortality, Emergency obstetrics services, SMS messaging, Rural area, Nigeria

## Abstract

**Background:**

Difficulty in transportation to access skilled providers has been cited repeatedly as a major barrier to utilization of emergency obstetric care in Nigeria.

**Objective:**

The objective of this paper is to describe the design, implementation, and outcomes of a mobile phone technology aimed at rapidly reaching rural Nigerian women who experience pregnancy complications with emergency transportation and access to providers.

**Method:**

The project was implemented in 20 communities in two predominantly rural Local Government Areas (LGAs) of Edo State, in southern Nigeria, as part of a larger implementation project aimed at improving the access of rural women to skilled pregnancy care. The digital health innovation named Text4Life, allowed women to send a brief message from their mobile phone to a server linked to Primary Health Care (PHC) facilities and to access pre-registered transport owners. Pregnant women were registered and taught to text short messages to a server from their mobile phones or those of a friend or relative when they experience complications.

**Results:**

Over 18 months, 56 women out of 1620 registered women (3.5%) texted the server requesting emergency transportation. Of this number, 51 were successfully transported to the PHC facilities, 46 were successfully treated at the PHC, and five were referred to higher-level care facilities. No maternal deaths occurred during the period, while four perinatal deaths were recorded.

**Conclusion:**

We conclude that a rapid short message sent from a mobile phone to a central server and connected to transport providers and health facility managers is effective in increasing the access of pregnant women to skilled emergency obstetric services in rural Nigeria.

## Introduction

Maternal mortality is a major public health challenge in developing countries, with a higher proportion of the burden among pregnant women living in rural communities. The World Health Organization (WHO) reports that 94% of the world’s 295,000 maternal deaths occur in low and lower-middle-income countries. Sub-Saharan Africa contributes 66% of these deaths [1]. Nigeria and India together account for about one-third of all global maternal deaths in 2017, with an estimated 67,000 deaths (23%) occurring in Nigeria [1]. According to the WHO, Nigeria was one of the countries that reduced its maternal mortality ratio (MMR) by less than 25% in response to the Millennium Development Goals (MDGs), while the majority of low-income and lower middle income reduced MMR by more than 25% [1]. The country’s MMR remains high with a lifetime risk of maternal death of 1 in 21 women [1].

A significant proportion of the Nigerian maternal deaths occur in rural communities and are linked to limited access to health services, weak health infrastructure, and adverse socio-cultural factors [2–6]. The 2018 Nigeria Demographic and Health Survey (NDHS) reported that overall, 67% of pregnant women accessed skilled prenatal services, about 43% accessed skilled delivery care, and as many as 72% of women in the rural Nigeria were attended at birth by unskilled providers [7]. Some of these rural women present in health facilities as un-booked emergencies and in most cases are too late for any meaningful emergency care to be implemented to avert death [6].

The Federal Ministry of Health (FMOH) and all major health policy agencies in Nigeria have recognized increased access to skilled obstetric care, especially in rural areas, as critical to reducing the high rate of maternal mortality. Although the Nigerian government acknowledges the critical role of primary health care in improving women’s access to skilled pregnancy care, especially in rural areas, the implementation and use of this model of care has been poor throughout the country [8, 9].

In 2017, we initiated formative, intervention, and implementation research to identify innovations and evidence for improving women’s access to skilled pregnancy care in Primary Health Centres (PHCs) in rural Nigeria. In the formative research, we identified difficulty with transportation as a major barrier to the use of existing PHCs by pregnant women [10, 11]. Through multiple qualitative research with women, men, and community leaders it became evident that women resorted to traditional methods of pregnancy care because of the lack of transportation when complications occurred, and unavailable providers at the facility, preferring to use unskilled providers such as unskilled traditional birth attendants (TBAs) that were easily accessible to them.

Maternal and child health initiatives across sub-Saharan Africa are embracing the use of electronic or mobile technology to improve access to and use of skilled obstetric care, particularly in marginalised and vulnerable populations. These initiatives have proven to be effective in improving women’s use of health facilities, in enhancing two-way communication between healthcare workers and pregnant women, and in increasing pregnant women’s self-efficacy through the provision of relevant health information [12–14].

Working in collaboration with women, community leaders, and policymakers and using community-based participatory research methods, we designed an intervention, Text4Life, which coordinates women with providers, transport, and supports triage of cases by the PHCs. The interventions were implemented for nearly 24 months, with impressive results, and with no recorded maternal mortality in the rural communities [15]. The objective of this paper is to describe the design and implementation of this technology and to reflect on its potentials for reducing the rate of maternal morbidity and mortality in rural communities.

## Methods

### Study design

This paper is drawn from a larger separate sample pretest-posttest quasi-experimental research conducted in rural Edo State, Nigeria between July 2017 and March 2020, and the post-project activities. The general aim of the larger research was to increase rural women’s access to skilled pregnancy care in primary healthcare centres.

### Study setting

The project was implemented in 20 randomly selected rural communities in two Local Government Areas (LGAs) of Edo State in Nigeria. Edo State is one of the 36 Federal states in Nigeria, with a population of over 4 million people, the majority of whom live in rural areas [16]. Edo State has 18 LGAs, each with at least 10 administrative wards, with 5000–10,000 people living in each ward.

The study was set in Etsako East and Esan South East LGAs, two predominantly rural LGAs located in the northern part of Edo State, both bordering the southern part of the River Niger as it enters the Atlantic Ocean. We chose Okpekpe in Etsako EastLGA and Ewatto in Esan South East LGA, two wards comprising 31 villages and hamlets, from which 20 were randomly selected for the project. Both wards have two PHCs each for four PHCs covering the villages and hamlets.

These communities were chosen because of their rural locations and the fact that PHCs are the only available sources of healthcare. There are no secondary or tertiary health care facilities in the immediate proximity although transfers can be made to secondary or tertiary facilities in other locations which are between 20 and 173 km.

### Study population and sample size

The sample size for the larger implementation research comprised 1408 ever married women at baseline and 1411 at endline who were randomly selected from households. The detailed description of the design, the selection of the study communities, sample size determination at the baseline and endline, and the intervention activities which included Rapidsms (Text4Life) have been described elsewhere [11, 15]. However, what is reported in this paper also includes pregnant women who registered in Text4Life after the larger project ended in March 2020.

### Formative research

The formative research included qualitative needs assessment to identify gaps and challenges. Women reported that the major challenges related to care were transportation difficulties and access to skilled providers, among others [10]. We then worked closely with community leaders where the use of rapid short message service (SMS) to link pregnant women to health providers was proposed as a solution. The plan to use mobile phones was considered viable because of the wide mobile phone usage in Nigeria. About 85% of the population in rural Nigeria have mobile phones [7], while the remaining with no phones often have access to those owned by their spouses, children, relatives, or friends. Thus, along with information communication technology (ICT) experts, we designed a rapid SMS model named Text4Life to be managed by members of the Ward Development Committees (WDC) in the communities.

Next, we worked with the local leaders to identify and appoint members of WDCs, with a chairperson for the two project sites and to develop and manage the application functions. The WDC is an initiative recommended by Nigeria’s Federal Ministry of Health to build community linkages and partnerships for the management of PHCs across the country [17]. Based on the formative research and subsequent brainstorming, we created Text4Life as a technology to establish real-time dual communication and alerts. The system was designed to run on an uninterrupted power supply, with a central database server located at the project office in Benin City, Nigeria. The system included a platform for the registration of new pregnancies with support to monitor pregnancies through the antenatal, delivery, and post-partum periods.

### Text4Life design and function

Text4Life was built upon an open-source framework for basic short message services, data collection, and communication platforms written in Python and Django programming language [18]. It was developed to enable instant reporting of pregnancy-related complications and timely notification of health facilities. The provider system ran on a desktop computer which served as the central server where all patient information is stored using a web user interface called “Textit”.

Textit receive messages from women and automatically sends dual replies to the phones of the WDC chairpersons and PHC workers. The device included a reversed billing method that triggers an alert message at no cost. We worked with the WDCs to identify reliable taxi owners in the communities who agreed to participate in the fully explained project. The technology included a web-user interface created for the project by Textit. This interface is a visual platform for interactive messaging. The account gives access to aggregated and disaggregated data for the team and enables the tracking of individual history of patients and reports. The password-protected web interface provides an overview of the system’s outputs, including individual messages sent out, reports, statistics, and administrative data.

The automated messages were designed to reply with customized messages (i.e., chatbots) that are then relayed to the sender, healthcare providers, and a WDC Chairman. This conversation workflow ran on an electronic communication device, looking out for SMS keywords and sending appropriate responses. Additionally, it can act on data from messages using Textit integrations and an application programming interface (API). It sends bulk SMS messages, managing them in an email-like inbox, sending automated messages, building bots for social networks, and keeping track of users in a simple customer relationship management (CRM) software.

In addition, each Textit® interaction is defined by a systematic workflow, which defines how the user of the application will progress through the flow. Textit® creates a logic flow routine to route users based on their responses. At any point in the flow, one can trigger an action, such as sending personalized short messages, emails, or calling through an external software intermediary such as the API, which can speed response time. Group messages are sent to pregnant women, the health workers, and WDC chairmen on a regular basis to provide health information and platform updates.

Each interaction creates a record associated with each user. The records are transferred into MS Excel representing data for all registered pregnant women. The user interface connects data across user groups for each project site, thereby serving as a tracker for the number of women registered and the inputs and outputs over the system. The system was designed to provide access to PHCs for pregnant women in case of an emergency. Text4life is accessed by pregnant women through registration with the WDC and the payment of a small fee - Naira 4000 (equivalent to less than $10) which could be paid in instalments. It enables the use of all facilities including antenatal, delivery, and postnatal care as well as the transportation at no cost. This was a part of a community health fund created as one of the intervention activities for the larger project [15].

### Text4Life implementation

**Fig. 1:**
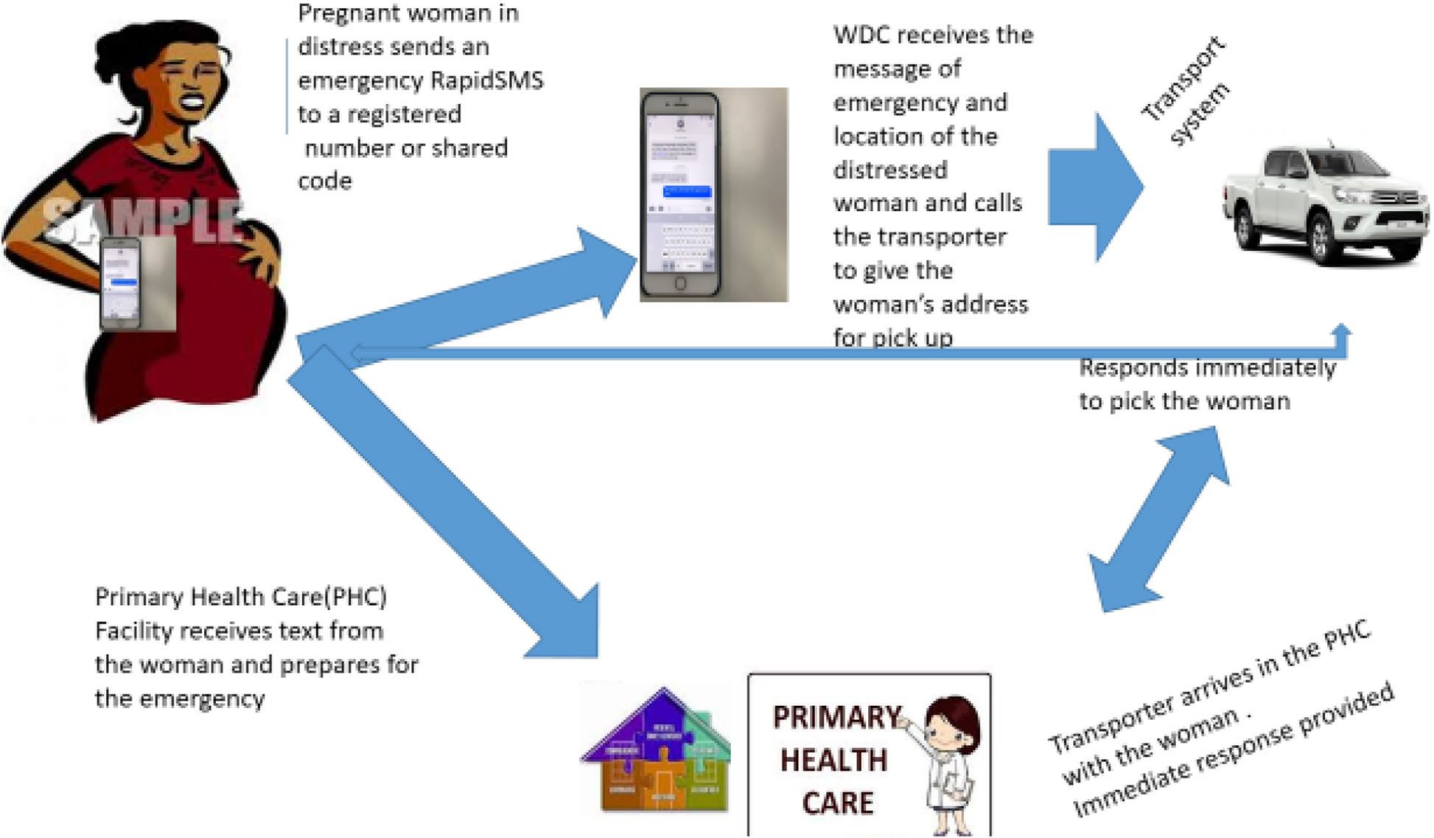
Summary of Text4life design and implementation

Women were recruited and registered, with records of contact details including their telephone numbers and those of their next of kin and neighbours. If a pregnant woman were in distress, she would trigger an alert system by sending a keyword to a dedicated registered phone number configured to the central server. The pregnant woman receives automated feedback from the server for her to wait while an action is being taken. Simultaneously, a dual SMS is relayed through a web-designed interface to the phone number of the WDC Chairman in the ward, and the health care provider at the PHC. Additionally, the pregnant woman’s information is displayed on the message relayed to the WDC and PHC, which prompts them to take immediate action. The WDC then calls the transport owner to pick up the woman in distress, while the healthcare providers prepare to receive the woman in the PHC (see Fig. 1).

The WDC Chairs oversee the project in their communities and report directly to the community leaders for timely decisions. The WDC educates community members about the need for antenatal clinics through village meetings. They also arrange the transport system, including referrals and transport to higher-level health facilities as needed. The WDC managed the community health fund, from which the cost of transportation and delivery care were paid.

### Training of WDC members, PHC providers, and community volunteers

To build capacity among community stakeholders to manage the system, a series of capacity-building workshops were organised. This consisted of four two-day workshops for the WDCs, PHC providers, and pregnant women (and their spouses or caregivers) in the two project sites (two per site) and two workshops for the health providers in the PHCs. During the workshops, trainers described the system and demonstrated the use of the Text4Life app. The education included explanation of the possible complications of pregnancy for which women would require immediate transfer. They were also taught the specific ways to send messages in English and interpret replies from the central server. Health providers were taught to prepare to receive women and the specific actions to be taken to respond to emergencies, including referrals to specific secondary care hospitals within the vicinity of the PHCs (with contact details) to which women with severe complications could be referred. On completion of the training, mobile phones were distributed to the healthcare workers in the PHCs and the WDC members. Each phone number was registered with all network providers in the communities.

The second training series was training of female volunteers in each community. These volunteers were selected based on literacy and ability to triage emergencies using the SMS system. Further trainings were conducted at each PHC.

### Analysis

Each participant and interaction are automatically uploaded to the server and transformed into variables. Additional variables were added to each record to denote outcomes of interactions and the pregnancy. Records were checked against WDC and PHC records to track the number of referrals to higher-level health facilities and the outcomes of treatment in the referral facilities.

Data were exported to Microsoft excel and analysed to review descriptive information about the number of women who registered in the project, number who reported complications using Text4Life, used the transport, treated in the PHCs or referred. The number of maternal and perinatal deaths was calculated.

## Results

The use of the device began in June 2019 and has continued since. The results presented here are for the period June 1, 2019, to December 31, 2020, spanning approximately 18 months of implementation.

As shown in Table 1, 1,620 pregnant women were registered in the two LGAs during the 18 months. There were slightly more registered women in Ewatto than in Okpekpe. The number of women reporting pregnancy complications to the Text4Life platform was 31 in Ewatto and 25 in Okpekpe for 56 women in both LGAs.

**Table 1:** Results of use of Text4life platform: June 2019 to December 2020

Indicator	Okpekpe	Ewatto	Total
Number of registered participants	774	846	**1620**
Number (%) reporting pregnancy complications in Text4Life	25 (3.2)	31 (3.7)	**56 (3.5)**
Number (%) transported to PHCs	23 (92.0)	28 (90.3)	**51 (91.1)**
Number (%) received and treated in PHCs	23 (100)	28 (100)	**51 (100)**
Number (%) referred to other facilities	2 (8.7)	3 (10.7)	**5 (9.8)**
Number (%) of maternal deaths	0 (0.0)	0 (0.0)	**0 (0.0)**
Number (%) of perinatal deaths	1 (0.1)	3 (0.4)	**4 (0.2)**

Ewatto (Esan Southeast) and Okpekpe (Etsako East) LGAs, Edo State

Of those reporting complications to the platform, 51 (91.1%) were transported by registered taxis to the PHCs. The common complications reported included bleeding, preterm labour, severe lower abdominal pain, and severe back pain among others. The reasons for five women not being transported included “taxi not available (on other duties) at the time” (4), while one woman delivered at home before the taxi arrived.

Among the 51 women transported to the PHCs, 46 were successfully treated, while five were referred and transferred by the same taxi to a referral hospital.

No maternal deaths were recorded among the women registered under the platform during the period, although four perinatal deaths occurred. Among the perinatal deaths, one was a macerated stillbirth in a woman who had foetal death in utero; two were stillbirths following labour at a secondary care hospital, while the remaining death was an early neonatal death due to preterm delivery.

## Discussion

This study described the implementation of a mobile phone app designed to rapidly reach rural pregnant women who experience complications with emergency transportation and access to providers. The results indicate that the intervention is feasible, especially when applied with the active participation of community members and transport services. Difficulty with transportation has featured repeatedly as one of the major challenges that limit women’s use of skilled pregnancy care especially in rural settings [10, 19–21]. While several interventions and innovations to address the bottlenecks have been proposed [22–26], this is one of a few such interventions that rely on the use of mobile phones. In this study, women who used the platform were successfully transported to the PHCs. While the majority were treated in the PHCs, a few were referred to higher level facilities, with no maternal deaths and four perinatal deaths reported.

We had also earlier reported the results of the post-intervention compared to the pre-intervention survey which showed a near elimination of transportation difficulty and provider unavailability as a cited barrier to the use of PHCs in the communities after the introduction of the text4life model [15]. The results of the study indicate what can be achieved in the prevention of maternal and perinatal deaths when a careful use of mobile phones is planned and implemented.

The use of technology in health has gained much attention in recent times [27]. With the continuous growth of mobile network coverage and unprecedented penetration of mobile devices in the developing world, several mobile health (eHealth) initiatives are being implemented to connect health workers and their patients, thereby improving the speed of decision-making and improving the lives of millions of underserved populations. In Uganda for example, an eHealth system has been used to manage the rollout of a malaria rapid diagnostic test by the country’s National Malaria Control Program [28]. Similarly, an SMS system to reduce delay in sending infant HIV testing results from a centralized laboratory to remote rural health facilities improved communication among health workers on family planning and reproductive health in rural areas in Zambia [29]. In addition, the Rwanda UNICEF eHealth project reported a 51% maternal mortality reduction from 750 per 100,000 live births estimates in 2005 to 476 per 100,000 live births by 2015 [12] These successes were reported to be associated with a comprehensive information technology strategy plan that included the use of combined electronic communication and information technology.

In Nigeria, while the use of electronic communication for registration of birth has been reported [30], there has been no evaluation on the use of mobile technology devices to improve maternal health services. Nigeria has a wide mobile phone usage with 89.1% of the population using mobile technology devices [7]. The wide coverage and use of mobile phones in Nigeria should provide an effective medium for increasing women’s access to skilled maternal care.

Several factors account for the success of this study. The first and the most important is the involvement and engagement of community stakeholders in the design and implementation of Text4Life. Well-selected WDC members that report periodically to community chiefs was critical to the project’s success as it ensured a high level of commitment to the project. This engagement process helped to build trust and accountability, which contributed to the project’s success. We believe that this approach will stimulate the sustainability of the project as key members of the community gained knowledge from the training and skills they received during the project implementation. Second, the project received garnered support from multi-stakeholders as described elsewhere [31], while the associated knowledge transfer helped to stimulate and sustain the interest of the public with expectations of positive project outcomes. Furthermore, the community benefitted from the information on optimal practices relating to maternal and child health that took place as part of the project activities.

This Text4Life app is different from other models. For example, in contrast to a project in Rwanda [12], our project enabled women in distress to trigger the SMS themselves, while the WDCs and health care workers are informed at the time the message is triggered. The Rwanda study required a community health worker to trigger the SMS. Our approach saves critical time. Text4Life averts the burden of keeping a health worker static in a position awaiting a call to trigger an SMS, and because its web interface is user-friendly, it can be easily installed on the mobile phone. Mobile phones allow for easy movement, communications, and monitoring of messages.

### Strengths and weaknesses

To the best of our knowledge, this is one of the most comprehensive eHealth technology innovation for increasing access to emergency skilled obstetrics care ever undertaken in the African region. The involvement of local community leaders ensured the project effectiveness and would likely promote its sustainability over time. The additional information provided on risks and methods of management of pregnancy during the project delivery also helped to expand the benefits of the project to include women’s agency and knowledge to use maternity services provided by skilled providers.

Despite these laudable outcomes, limitations of concern include the fact that only 56 women out of the 1620 registered pregnant women (3.5%) used the Text4life platform for emergency transportation. This may be because the use of the platform was recommended for women who lived far away from the PHCs and who had no immediate transport at the time they experienced a complication. Our record indicates that among women requesting transportation through the Text4Life platform, a large proportion (> 90%) were moved to the PHCs with emergency transport. Among those not reached with transport, this was due to late arrival or non-availability of the taxis at the time the request was made. This implies that a system involving the use of multiple and alternative methods of transport and that includes a response from the transport owners regarding their availability at the time of the request would have to be considered.

In addition, the study is limited by its restricted scope given that only two wards in two LGAs were included in the sample. A research design based on a larger sample size involving several sites in different parts of the country would be necessary to identify the challenges and bottlenecks that need to be overcome for scaling the innovation throughout Nigeria’s health care system. This may perhaps include a more systematic randomised or quasi-experimental research designed in the format of implementation research to provide information on the appropriate context for the delivery of the intervention in the country.

## Conclusion

In conclusion, this study demonstrates the potential of a rapid short message sent from a mobile phone to a central server and connected to transport providers and health facility managers to increase the access of pregnant women to skilled emergency obstetric services in rural Nigeria. Further study with a larger sample of women and covering wider population segments will be needed to firmly establish this approach as effective for reducing transportation delay as a cause of the high rate of maternal mortality in Nigeria.

## Data Availability

The data presented in this paper can be accessed on request from the corresponding author. The larger dataset for the intervention can be accessed from OpenICPSR 10.3886/E123302

## Abbreviations

WHO: World Health Organization
MDGs: Millennium Development Goals
MMR: Maternal Mortality Ratio
NDHS: Nigeria Demographic and Health Survey
FMOH: Federal Ministry of Health
PHC: Primary Health Centre
TBA: Traditional Birth Attendant
LGA: Local Government Area
SMS: Short Message Service
ICT: Information Communication Technology
WDC: Ward Development Committee
API: Application Programming Interface
CRM: Customer Relationship Management
